# Comparative Chloroplast Genomics Reveals Phylogenetic Relationships Among Korean *Dioscorea* Species

**DOI:** 10.1002/ece3.74651

**Published:** 2026-11-08

**Authors:** Ji Eun Kim, Min Su Jo, Kyung‐Bae Nam, Jun Hyeok Kim, Jun‐Ho Song, Hyeok‐Jae Choi, Gyu Young Chung, Chae Sun Na

**Affiliations:** ^1^ Wild Plant Seed Division Baekdudaegan National Arboretum Bonghwa‐gun Gyeongsangbuk‐do Republic of Korea; ^2^ Department of Biology Chungbuk National University Cheongju‐si Chungcheongbuk‐do Republic of Korea; ^3^ Department of Biology and Chemistry Changwon National University Changwon‐si Gyeongsangnam‐do Republic of Korea; ^4^ Department of Forest Science Gyeongkuk National University Andong‐si Gyeongsangbuk‐do Republic of Korea

**Keywords:** chloroplast, crop wild relatives, *Dioscorea*, phylogenetic relationship

## Abstract

Crop wild relatives represent important sources of genetic variation for crop improvement, particularly as climate change and increasing global food demand require the development of more resilient and productive crop varieties. However, genomic information on many wild *Dioscorea* species in East Asia remains limited. In this study, we sequenced and assembled the complete chloroplast genomes of six *Dioscorea* species native to Korea, including the first reported complete chloroplast genome of *D. coreana*. The assembled genomes exhibited a conserved quadripartite structure with similar gene contents and GC compositions across species. Despite this overall conservation, comparative analyses revealed localized sequence variation, particularly in noncoding regions. Several loci, including *ndhF* and *ycf1*, exhibited increased nucleotide diversity, indicating their potential as informative markers. In addition, simple sequence repeats were composed mainly of A/T‐rich motifs and predominantly distributed in the large single‐copy region. Phylogenetic analysis based on concatenated chloroplast protein‐coding gene sequences provided a well‐resolved framework for the studied taxa and clarified the relationships among Korean *Dioscorea* species. Furthermore, the close genetic relationship between cultivated yam and its wild relatives was supported. These results provide new genomic data for previously underrepresented Korean taxa, including the first chloroplast genome of *D. coreana*, and offer a basis for future studies on genetic diversity, species identification, and the utilization of wild germplasm.

## Graphical abstract

Comparative analysis of the complete chloroplast genomes of six Korean *Dioscorea* species revealed highly conserved genome structures with localized sequence variation, including highly variable regions in *ndhF* and *ycf1*. Phylogenetic analysis based on chloroplast protein‐coding sequences clarified relationships among Korean *Dioscorea* species and their relatives, providing genomic resources for future studies of species identification, genetic diversity, and wild germplasm.

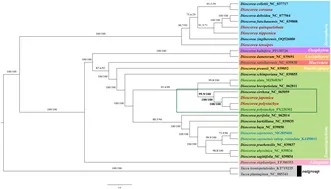

## 1 Introduction

Global agriculture is increasingly threatened by climate change and the demands of a growing population, which together place substantial pressure on food production systems (Cockel et al. 2022; Begna and Wakweya 2025; Qu et al. 2026). Enhancing the resilience of crop species has therefore become a critical priority for modern agriculture. One promising strategy involves the utilization of genetic diversity found in crop wild relatives (CWRs), which are wild plant species closely related to cultivated crops (Zhang et al. 2017; Tirnaz et al. 2022; Kapazoglou et al. 2023). These species represent important reservoirs of genetic variation and provide valuable traits for crop improvement (Hajjar and Hodgkin 2007; Balla et al. 2022; Rajan et al. 2025). As a result, CWRs are increasingly recognized as important genomic resources for crop improvement and sustainable agricultural development.

Yams (*Dioscorea* spp.) are among the most important root and tuber crops cultivated in tropical and subtropical regions, where they serve as staple foods for millions of people and contribute significantly to regional food security (Karnick 1969; Zannou et al. 2004; Asiedu and Sartie 2010). Globally, yams are among the major root and tuber crops, following potato ( *Solanum tuberosum* ), cassava ( *Manihot esculenta* ), and sweet potato ( *Ipomoea batatas* ) (Scott 2021). Yam tubers are rich in carbohydrates, dietary fiber, vitamins, and minerals, making them an important nutritional resource in many developing countries (Govaerts et al. 2007; Polycarp et al. 2012; Otegbayo et al. 2014). In addition to their value as staple foods, several yam species are recognized for their medicinal properties (Dutta 2015; Obidiegwu et al. 2020).

Because of their economic, nutritional, and medicinal importance, yams have attracted increasing attention in agricultural research and crop improvement programs (Chu and Figueiredo‐Ribeiro 1991; Dutta 2015; Darkwa et al. 2020). Yams belong to the genus *Dioscorea* L. (Alexander and Coursey 1969), the largest genus within the family Dioscoreaceae, which comprises approximately 600 species distributed across tropical and subtropical regions, as well as parts of East Asia, the Mediterranean Basin, and the Americas (Coursey 1967; Obidiegwu et al. 2020). While only a limited number of *Dioscorea* species have been domesticated (Scarcelli et al. 2017), numerous wild *Dioscorea* species occur naturally across diverse habitats and may represent important sources of genetic diversity within the genus. This diversity is considered a potentially valuable resource for understanding evolutionary relationships and for supporting future yam improvement efforts.

In East Asia, including Korea, several wild *Dioscorea* species occur naturally and contribute to the genetic diversity of this genus. However, the phylogenetic relationships among many of these species remain insufficiently resolved. In Korea, approximately eight species of *Dioscorea*, including the wild taxon *D. coreana*, have been reported (Jeong et al. 2020, 2022; Chung and Chung 2016). Many of these species are regarded as CWRs of cultivated yams and may represent valuable genetic resources for breeding and crop improvement. Although Korean *Dioscorea* species are not currently included in the GRIN‐Global CWR inventory, several species have traditionally been consumed as edible plants and may represent potential wild relatives of cultivated yams. Although complete chloroplast genomes have been reported for several *Dioscorea* species (Wu et al. 2016; Feng, Zhang, et al. 2024; Feng, Zheng, Jiang, et al. 2024), comparative genomic information on Korean accessions remains limited. In particular, the complete chloroplast genome of *D. coreana* has not previously been reported, and the phylogenetic relationships among Korean *Dioscorea* taxa and cultivated yams warrant further investigation. This lack of genomic information limits our understanding of the genetic diversity and evolutionary relationships of Korean *Dioscorea* species, including their relationships with cultivated yams.

To better understand the evolutionary and phylogenetic relationships between cultivated yams and their wild relatives, genomic resources that provide informative variation among *Dioscorea* species are needed. Chloroplasts, key organelles involved in photosynthesis, energy conversion, and metabolic regulation in plants, are present in land plants, algae, and some protozoa (Woodson and Chory 2008; Wicke et al. 2011; Bhattacharyya and Chakraborty 2018; Feng, Zhang, et al. 2024; Feng, Zheng, Jiang, et al. 2024). Chloroplast genomes are typically 107–218 kb in size and contain approximately 100–120 genes, with a conserved quadripartite structure composed of long single‐copy (LSC), small single‐copy (SSC), and two inverted repeat (IR) regions (Wicke et al. 2011). Owing to their conserved genomic structure and relatively slow evolutionary rate, chloroplast genomes have become widely used as molecular resources for investigating evolutionary relationships in plants (Daniell et al. 2016). Comparative analyses of chloroplast genomes can provide insights into genome evolution, species divergence, and phylogenetic relationships among closely related taxa (Xu et al. 2022; Qin et al. 2025; Xiao et al. 2025). In the context of CWR research, understanding phylogenetic relationships between wild and cultivated species represents an important first step toward evaluating wild relatives as potential genetic resources. Chloroplast genome sequences have been widely used to infer phylogenetic relationships in plants and provide valuable insights into the evolutionary origins of cultivated species and their wild relatives (Daniell et al. 2016). Well‐resolved phylogenetic frameworks can clarify evolutionary relationships and patterns of genetic diversity within a genus and may also provide a basis for evaluating the relationships between cultivated species and their wild relatives.

In this study, we sequenced and comparatively analyzed the complete chloroplast genomes of six *Dioscorea* species native to Korea, including the first reported complete chloroplast genome of *D. coreana*. We aimed to (i) characterize chloroplast genome structure and organization, (ii) identify patterns of sequence variation and candidate molecular markers, and (iii) clarify the phylogenetic relationships of Korean *Dioscorea* species in relation to cultivated yams and other closely related taxa. Our findings expand the genomic resources available for Korean *Dioscorea* and provide a foundation for future studies of their systematics, genetic diversity, and relationships with cultivated yams.

## 2 Materials and Methods

### 2.1 Plant Materials

In this study, six *Dioscorea* species from Korea were included in the chloroplast genome analysis (Figure 1). Leaf samples were collected from voucher specimens obtained from natural populations in different regions of South Korea and used for genomic DNA extraction. The sampling sites were selected from populations represented by seed accessions conserved in the Baekdudaegan National Arboretum Seed Bank with corresponding voucher specimens deposited in the herbarium (Table 1). The seed accessions correspond to five species: *D. japonica* , *D. nipponica* , *D. polystachya* , *D. quinqueloba*, and *D. coreana*. For *D. tenuipes* , no associated seed accession was available; instead, the voucher specimen is preserved in the herbarium of the Baekdudaegan National Arboretum.

**FIGURE 1:**
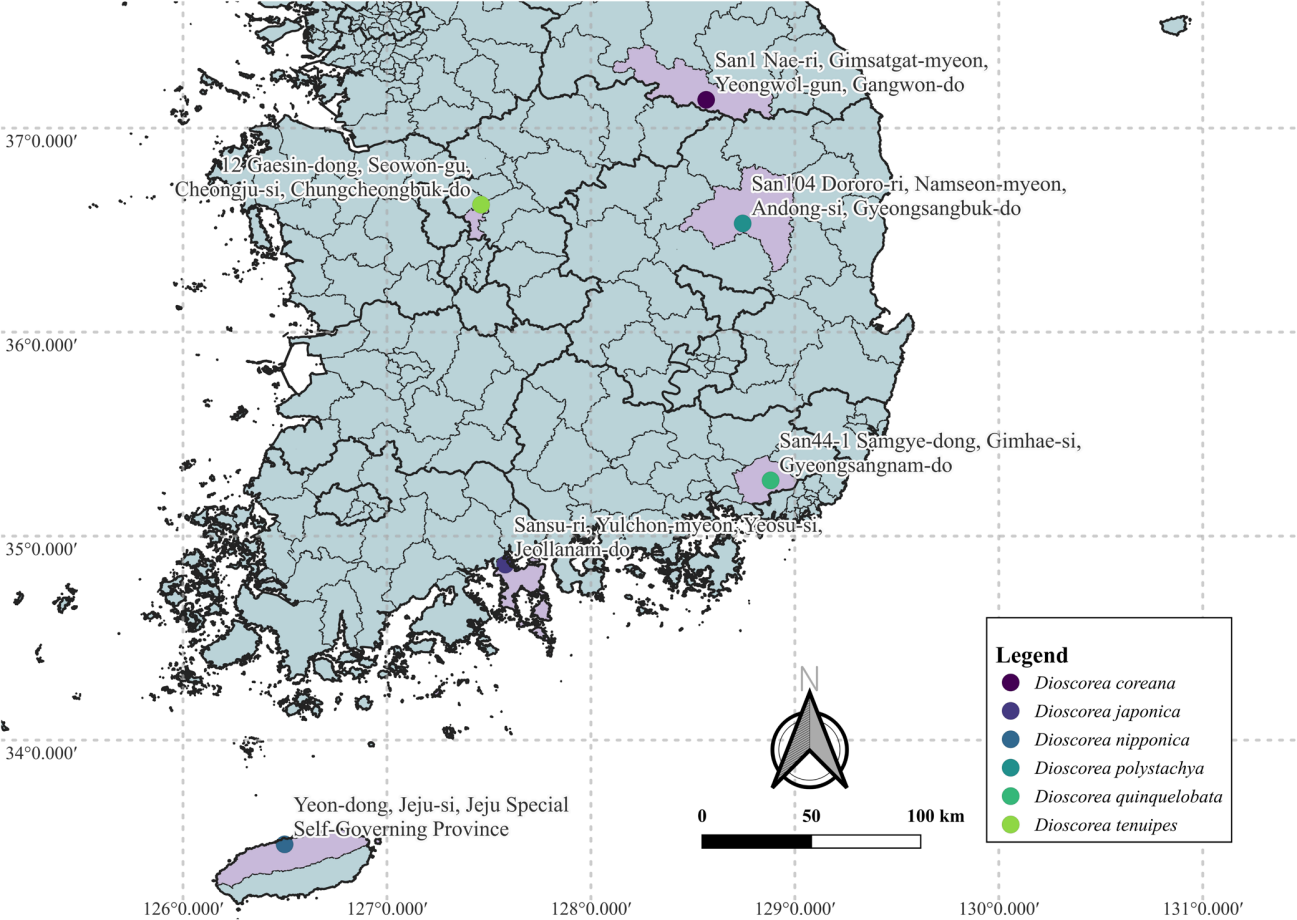
Map showing the collection sites of the six *Dioscorea* species included in this study. The sampling locations are labeled with abbreviated locality information.

**TABLE 1:** List of *Dioscorea* samples with corresponding voucher and DNA sample numbers.

No.	Scientific name	Accession no.	
		Voucher	DNA
1	*D. coreana*	2023‐047550	BD003572
2	*D. japonica*	2023‐047556	BD003573
3	*D. nipponica*	2023‐047559	BD003575
4	*D. polystachya*	2023‐047562	BD003571
5	*D. quinqueloba*	2023‐047577	BD003576
6	*D. tenuipes*	241029‐ANU‐P002	BD003574

### 2.2 DNA Extraction and Genome Assembly Procedures

Total genomic DNA was extracted from fresh leaves using a NucleoSpin Plant II Kit (Macherey‐Nagel; Düren, Germany) following the manufacturer's instructions. DNA quality and concentration were assessed using a NanoDrop 2000 spectrophotometer (Thermo Fisher Scientific; Wilmington, DE, USA) and a Qubit 3.0 fluorimeter (Invitrogen; Carlsbad, CA, USA). Paired‐end libraries were prepared using the TruSeq Nano DNA Library Preparation Kit (Illumina; San Diego, CA, USA) and sequenced on the Illumina NovaSeq platform.

For each sample, approximately 2.5–3.3 Gb of raw sequencing data (16.2–22.1 million paired‐end reads) were generated, providing more than 100× coverage of the chloroplast genome. Raw reads were processed by removing adapter sequences and filtering out low‐quality reads with a Phred score < 20. The chloroplast genomes were assembled de novo using CLC Assembly Cell v4.2.1 (CLC Inc.; Aarhus, Denmark). Genome annotation was conducted using GeSeq, and the annotations were manually verified and corrected in Geneious Prime (Biomatters Ltd.; Auckland, New Zealand). A circular representation of the chloroplast genome was generated using OGDRAW v. 1.3.1 (Wu et al. 2025). The complete chloroplast genome sequences obtained in this study have been deposited in the GenBank database (PZ211770–PZ211775) and the Korea Biodiversity Data System (K‐BDS).

### 2.3 Comparative Analysis of Chloroplast Genomes in Dioscorea Species

Codon usage patterns were evaluated by calculating relative synonymous codon usage (RSCU) values from protein‐coding sequences using MEGA v12.0.11 (Jia and Higgs 2008; Kumar et al. 2024). RSCU values describe the relative frequency of synonymous codons encoding the same amino acid. A value lower than 1 indicates that a codon is used less frequently than expected, a value close to 1 suggests no apparent bias, and a value greater than 1 indicates preferential usage (Sobreiro et al. 2020; Chen et al. 2024). In this study, codons with an RSCU value < 0.6 were regarded as extremely rare, whereas those with an RSCU value greater than 1.6 were considered strongly favorable (Wright 1990; Wong et al. 2010; Shi and Xia 2019). Simple sequence repeats (SSRs) were detected using the MISA‐web platform (https://webblast.ipk‐gatersleben.de/misa/; accessed March 19, 2025). The minimum repeat threshold was set to 10 for mononucleotide motifs; 5 for dinucleotides; 4 for trinucleotides; and 3 for tetra‐, penta‐, and hexanucleotide repeats. In addition, REPuter was used to identify long repetitive sequences, including dispersed (D) and palindromic (P) repeats, with a minimum repeat length of 20 bp and a maximum Hamming distance of three (https://bibiserv.cebitec.uni‐bielefeld.de/reputer; accessed March 19, 2025). The boundaries of the IR regions among the six *Dioscorea* species were visualized using the IRscope web server (Amiryousefi et al. 2018) (https://irscope.shinyapps.io/irapp/; accessed March 18, 2026). To evaluate nucleotide diversity (π) across chloroplast coding regions, protein‐coding sequences from the six *Dioscorea* species were aligned and concatenated, and sliding window analysis was performed in DnaSP v5 using a window length of 400 bp and a step size of 200 bp (Librado and Rozas 2009). Multiple sequence alignment was carried out using MAFFT v7.490 (Katoh and Standley 2013), followed by manual inspection and editing in Geneious Prime v2025.1.2. Sequence divergence among the six *Dioscorea* species was further assessed using the mVISTA platform (http://genome.lbl.gov/vista/mvista/submit.shtml; accessed March 19, 2025). Alignments were generated using the LAGAN algorithm (Brudno et al. 2003), with the chloroplast genome of *D. cayenensis* (GenBank accession number: MG805606) used as the reference sequence. The comparative genomic analyses focused on the six newly sequenced Korean *Dioscorea* accessions, as the primary objective was to characterize these genomic resources rather than to conduct an exhaustive genus‐wide or intraspecific comparison.

### 2.4 Phylogenetic Relationships Among Dioscorea Species and Cultivated Yams

To investigate the phylogenetic relationships within *Dioscorea*, complete chloroplast genome sequences representing 22 *Dioscorea* species and 2 *Tacca* species were obtained from the NCBI GenBank database (https://www.ncbi.nlm.nih.gov/, accessed October 28, 2025). Species classification, including CWRs, landraces, and cultivated species, followed the GRIN‐Global distribution data (Postman et al. 2010; Trinder et al. 2024). Protein‐coding genes were extracted using PhyloSuite v1.2.3 (Xiang et al. 2023) and subsequently aligned with MAFFT v7.490 (Katoh and Standley 2013). The aligned sequences were concatenated into a single dataset and manually inspected in Geneious Prime v2025.1.2. Maximum likelihood (ML) phylogenetic reconstruction was performed in IQ‐TREE using the concatenated dataset. The best‐fitting substitution model selected according to the Bayesian information criterion (BIC) was GTR + F + I + G4. Branch support was evaluated using 1000 SH‐like approximate likelihood ratio test (SH‐aLRT) replicates and 1020 ultrafast bootstrap (UFBoot) replicates.

## 3 Results

### 3.1 Conserved Chloroplast Genome Structure Among Dioscorea Species

To characterize and compare the overall chloroplast genome organization of the six *Dioscorea* species, genome size, quadripartite structure, gene content, and GC composition were examined. The complete chloroplast genomes of the six *Dioscorea* species exhibited a high degree of similarity in terms of genome organization, gene content, and GC composition (Figure 2, Table 2). The genome sizes ranged from 153,219 to 154,048 bp. All the genomes exhibited the quadripartite structure typical of angiosperm chloroplast genomes, consisting of an LSC region (83,403–84,075 bp), an SSC region (18,821–18,938 bp), and a pair of IR regions (25,487–25,580 bp). The overall GC content ranged from 37.01% to 37.20% among the six species. This relatively consistent GC composition parallels the AT‐rich nature common to chloroplast genomes. A higher GC content, ranging from 42.9% to 43.0%, was observed in the IR regions than in the LSC (34.8%–35.0%) and SSC regions (30.9%–31.2%).

**FIGURE 2:**
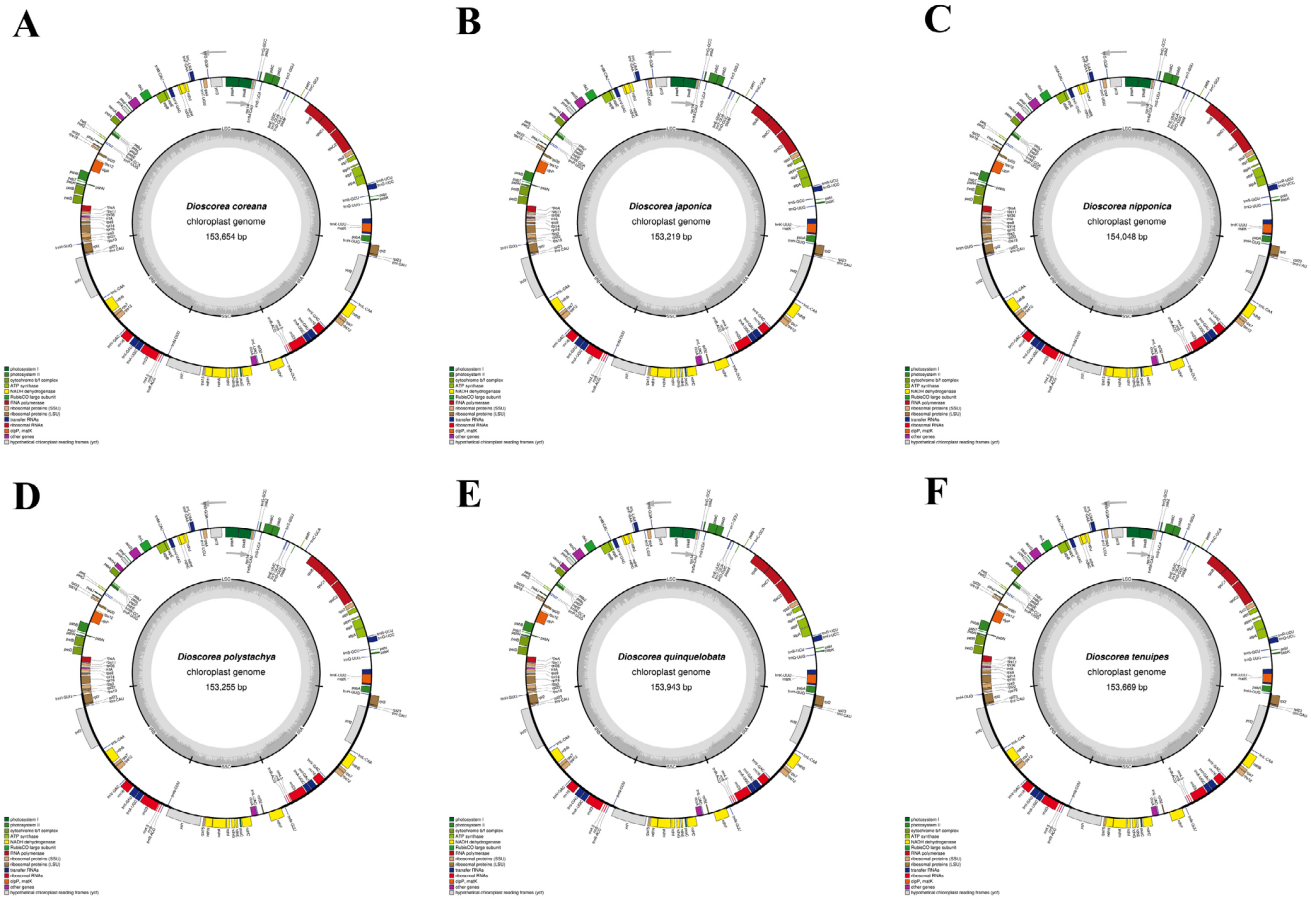
Circular maps of the chloroplast genomes of the six *Dioscorea* species analyzed in this study. Functional gene groups are indicated by different colors. Arrows represent the direction of gene transcription, with genes on the outer ring transcribed clockwise and those on the inner ring transcribed counterclockwise. The gray area in the inner circle represents variation in GC content across the chloroplast genome.

**TABLE 2:** Summary of complete chloroplast genome features of six *Dioscorea* species: (A) *D. coreana*, (B) *D. japonica* , (C) *D. nipponica* , (D) *D. polystachya* , (E) *D. quinqueloba*, and (F) *D. tenuipes* .

Attribute	A	B	C	D	E	F
NCBI	PZ211770	PZ211771	PZ211772	PZ211773	PZ211774	PZ211775
K‐BDS	SN01002273	SN01002272	SN01002274	SN01002275	SN01002270	SN01002271
Total length (bp)	153,654	153,219	154,048	153,255	153,943	153,669
LSC length (bp)	83,612	83,403	84,075	83,456	83,975	83,595
SSC length (bp)	18,938	18,822	18,913	18,821	18,902	18,914
IR length (bp)	25,552	25,497	25,530	25,489	25,533	25,580
Total GC (%)	37.20	37.01	37.19	37.02	37.19	37.19
LSC GC (%)	35.0	34.8	35.0	34.8	35.0	35.0
SSC GC (%)	31.2	30.9	31.2	31.0	31.2	31.1
IR GC (%)	43.0	42.9	43.0	42.9	43.0	43.0
Total genes	130 (112)	130 (112)	130 (112)	130 (112)	130 (112)	130 (112)
CDS	84 (78)	84 (78)	84 (78)	84 (78)	84 (78)	84 (78)
tRNA	38 (30)	38 (30)	38 (30)	38 (30)	38 (30)	38 (30)
rRNA	8 (4)	8 (4)	8 (4)	8 (4)	8 (4)	8 (4)

*Note:* Numbers in parentheses indicate the counts of unique genes, excluding duplicated copies located in the IR regions.

Abbreviations: CDS, coding sequence; GC, guanine‐cytosine; IR, inverted repeat; K‐BDS, Korea BioData Station; LSC, large single‐copy; NCBI, National Center for Biotechnology Information; rRNA, ribosomal RNA; SSC, small single‐copy; tRNA, transfer RNA.

A total of 130 genes, including 84 protein‐coding genes, 38 tRNA genes, and 8 rRNA genes, were annotated in each chloroplast genome when duplicated copies in the IR regions were included (Tables 2 and 3). Excluding these duplicated copies, each genome contained 112 unique genes, comprising 78 protein‐coding genes, 30 tRNA genes, and 4 rRNA genes. These genes were classified into 18 functional groups. Among these genes, six protein‐coding genes (*ycf2*, *rps7*, *rps12*, *rpl2*, *rpl23*, and *ndhB*), eight tRNA genes (*trnN*‐*GUU*, *trnR*‐*ACG*, *trnA*‐*UGC*, *trnI*‐*GAU*, *trnV*‐*GAC*, *trnL*‐*CAA*, *trnI*‐*CAU*, and *trnH*‐*GUG*), and four rRNA genes (*rrn16*, *rrn23*, *rrn4.5*, and *rrn5*) were present in duplicate within the IR regions. The *rps12* gene exhibited a trans‐splicing structure, with the 5′ exon located in the LSC region and the 3′ exon located in the IR region. In addition, the *matK* gene was embedded within the intron of *trnK*‐*UUU*.

**TABLE 3:** List of genes annotated in the chloroplast genomes of six *Dioscorea* species.

Gene function	Gene symbols
Subunits of ATP synthase	*atpA, atpB, atpE, atpF, atpH, atpI*
Subunits of NADH‐dehydrogenase	ndhA, ndhB*, *ndhC, ndhD, ndhE, ndhF, ndhG, ndhH, ndhI, ndhJ, ndhK*
Subunits of cytochrome b/f complex	*petA, petB, petD, petG, petL, petN*
Subunits of photosystem I	*psaA, psaB, psaC, psaI, psaJ*
Subunits of photosystem II	*psbA, psbB, psbC, psbD, psbE, psbF, psbH, psbI, psbJ, psbK, psbL, psbM, psbN, psbT, psbZ*
Large subunit of ribosome	rpl14, rpl16, rpl2*, rpl20, rpl22, rpl23*, *rpl32, rpl33, rpl36*
Small subunit of ribosome	rps11, rps12*, rps14, rps15, rps18, rps19, rps2, rps3, rps4, rps7*, *rps8*
DNA dependent RNA polymerase	*rpoA, rpoB, rpoC1, rpoC2*
Subunit of rubisco	*rbcL*
c‐type cytochrome synthesis gene	*ccsA*
Envelop membrane protein	*cemA*
Maturase	*matK*
Protease	*clpP*
Subunit of Acetyl‐CoA‐carboxylase	*accD*
Translational initiation factor	*infA*
Conserved open reading frames	ycf1, ycf2*, *ycf3, ycf4*
Transfer RNAs	*trnK*‐*UUU, trnQ*‐*UUG, trnS*‐*GCU, trnG*‐*UCC, trnR*‐*UCU, trnC*‐*GCA, trnD*‐*GUC, trnY*‐*GUA, trnE*‐*UUC, trnT*‐*GGU, trnS*‐*UGA, trnG*‐*GCC, trnfM*‐*CAU, trnS*‐*GGA, trnT*‐*UGU, trnL*‐*UAA, trnF*‐*GAA, trnV*‐*UAC, trnM*‐*CAU, trnW*‐*CCA, trnP*‐*UGG, trnH*‐GUG*, *trnI*‐CAU*, *trnL*‐CAA*, *trnV*‐GAC*, *trnI*‐GAU*, *trnA*‐UGC*, *trnR*‐ACG*, *trnN*‐GUU*, *trnL*‐*UAG*
Ribosomal RNAs	rrn16*, rrn23*, rrn4.5*, rrn5*

Abbreviation: Gene*, genes present in two copies in the chloroplast genome.

### 3.2 Conserved A/U‐Biased Codon Usage in Dioscorea Chloroplast Genomes

To characterize patterns of synonymous codon usage and assess whether these patterns were conserved among the six *Dioscorea* chloroplast genomes, RSCU values were compared across protein‐coding genes. RSCU was calculated on the basis of the protein‐coding sequences of the chloroplast genomes from six *Dioscorea* species, encompassing 64 codons corresponding to 20 amino acids including stop codons (Figure 3). Across the genus, codon usage patterns were highly similar, with minor variations in RSCU values among species. Leucine (Leu), encoded by six synonymous codons (UUA, UUG, CUU, CUC, CUA, and CUG), was the most frequently represented amino acid and was associated with the highest overall codon abundance. Among the synonymous codons, the RSCU values for those ending with A or U were generally higher, indicating a clear preference for codons ending with A/U in the chloroplast genomes. In particular, the RSCU values for several codons, such as UUA (Leu), UCU (Ser), ACU (Thr), and GCU (Ala), were relatively elevated, suggesting strong codon usage bias. In contrast, codons with G or C at the third position tended to have lower RSCU values, reflecting underrepresentation. Consistent with the features typical of the chloroplast genome, AUG (Met) and UGG (Trp), each encoded by a single codon, exhibited RSCU values close to 1, indicating no codon usage bias. Overall, approximately half of the codons had RSCU values greater than 1, whereas the remaining codons had values less than 1, indicating a balanced but biased codon usage pattern. Overall, the similar RSCU profiles among the six species indicate a highly conserved pattern of chloroplast codon usage, characterized predominantly by a preference for A‐ or U‐ending synonymous codons.

**FIGURE 3:**
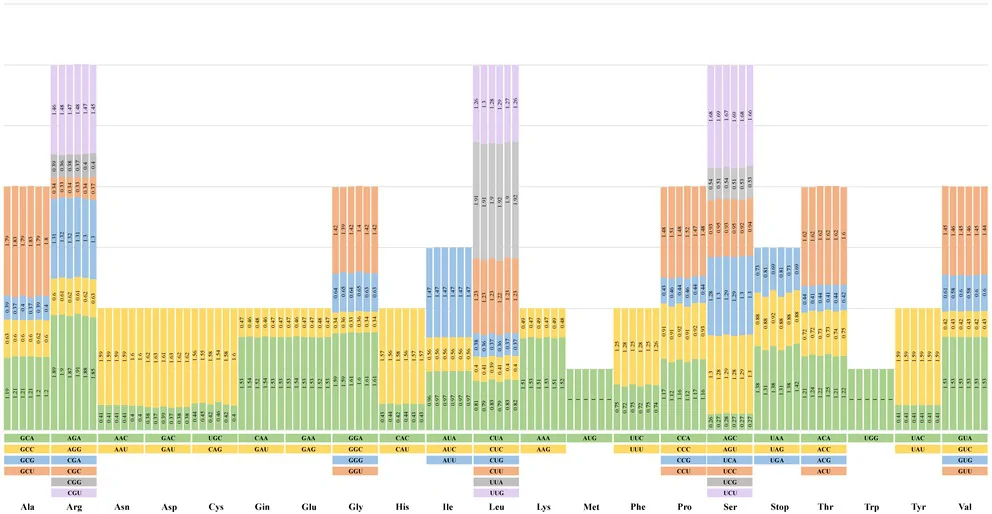
Relative synonymous codon usage (RSCU) patterns in the chloroplast genomes of six *Dioscorea* species. Codons are arranged along the *x*‐axis according to their corresponding amino acids, and the *y*‐axis represents RSCU values. For each codon, six adjacent bars correspond to six species in the following order from left to right: (1) *D. coreana*, (2) *D. japonica* , (3) *D. nipponica* , (4) *D. polystachya* , (5) *D. quinqueloba*, and (6) *D. tenuipes* . The colors indicate individual codons, as shown in the legend.

### 3.3 Conserved Patterns of SSRs and Long Repeats Among Dioscorea Species

To examine patterns of repetitive sequence variation among the six *Dioscorea* chloroplast genomes, SSRs and long repeat sequences were identified and compared. The occurrence and distribution of SSRs were examined across the chloroplast genomes of six *Dioscorea* species (Figure 4). A total of 71–84 SSR loci were identified per genome, with *D. tenuipes* exhibiting the greatest number (84) and *D. polystachya* the lowest (71) (Figure 4A). Among the SSR types, mononucleotide repeats were the most abundant, comprising the majority of the detected motifs (38–49 loci), followed by dinucleotide repeats (12–17 loci). Trinucleotide repeats were relatively rare, with 4–10 occurrences, whereas tetra‐, penta‐, and hexanucleotide repeats were relatively rare across all species. In terms of regional distribution, SSRs were predominantly located in the LSC region, where the highest counts were consistently observed (51–61 loci). The SSC region contained a moderate number of SSRs (12–19 loci), whereas both inverted repeat regions (IRa and IRb) contained relatively few SSRs, typically 3–4 loci each (Figure 4B). Analysis of repeat motif composition revealed a strong bias toward A/T‐rich sequences. The mononucleotide repeats were largely composed of A/T motifs, and this trend was also evident in longer repeat units. Overall, SSR motifs containing A or T bases predominated across all species, whereas GC‐rich repeats were relatively infrequent (Figure 4C).

**FIGURE 4:**
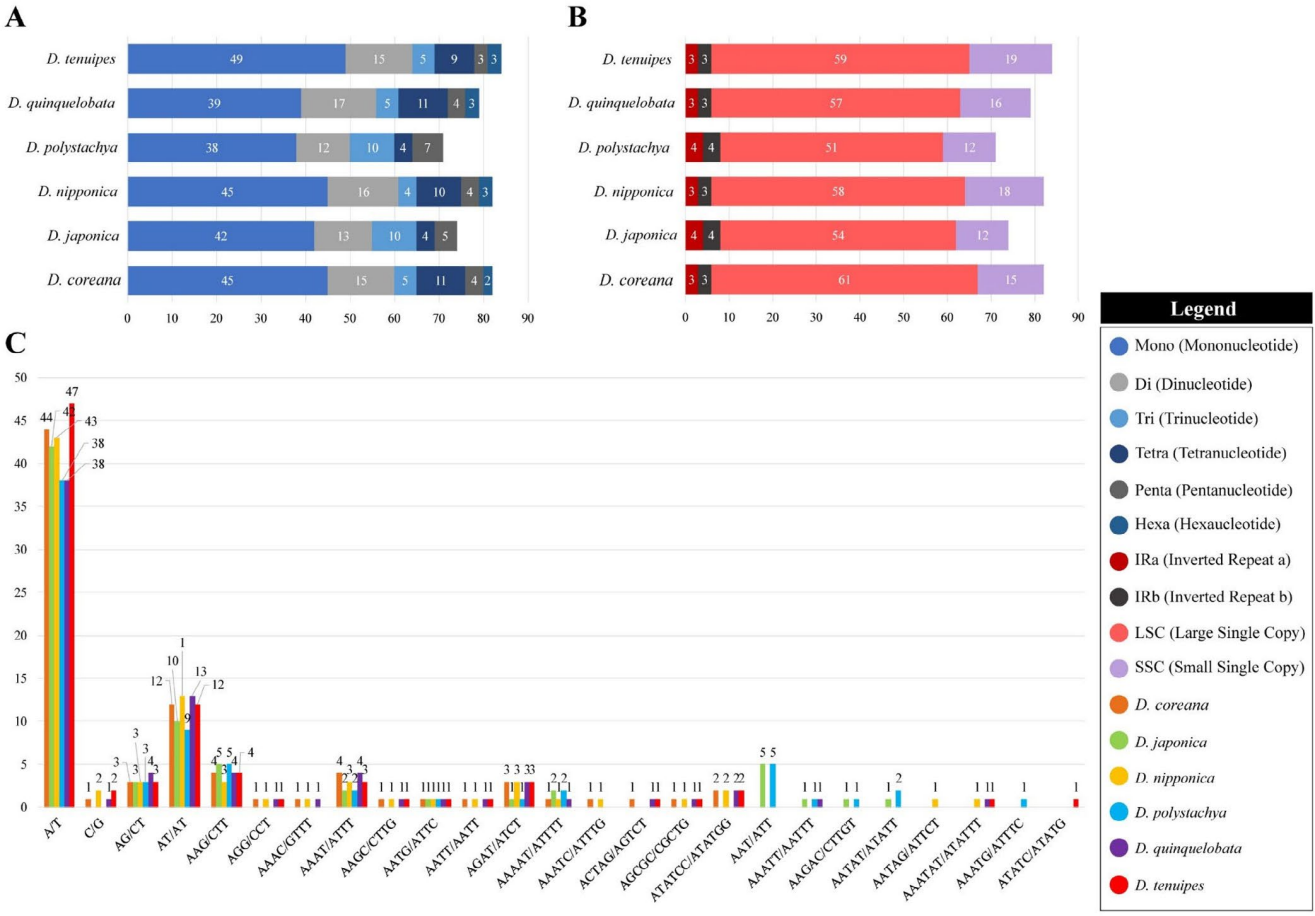
Composition and distribution of chloroplast simple sequence repeats in six *Dioscorea* species. The x‐axis denotes SSR categories and species, while the y‐axis shows the number of identified SSRs. (A) Overall abundance and classification of SSR types. (B) Distribution of SSRs across different genomic regions. (C) Proportional representation of SSRs based on repeat unit categories.

To characterize and compare long‐repeat patterns among the six *Dioscorea* chloroplast genomes, the type, length, and abundance of long‐repeat sequences were examined. Analysis of long‐repeat sequences in the chloroplast genomes of six *Dioscorea* species revealed that palindromic (P) repeats were more abundant than dispersed (D) repeats across all taxa, whereas other repeat types were not detected (Figure 5). Most long‐repeat sequences were concentrated in the 30–39 bp length class, which represented a size category predominant in all species (Figure 5A). In contrast, repeats exceeding 50 bp were infrequent and occurred only in limited numbers. Although the overall distribution patterns across species were similar, minor variations in repeat abundance were observed. A total of 31–40 long‐repeat sequences were identified per genome, comprising 13–17 dispersed repeats and 17–23 palindromic repeats (Figure 5B). Among the examined species, *D. coreana* and *D. japonica* exhibited relatively high numbers of repeat sequences, whereas *D. nipponica* and *D. tenuipes* exhibited relatively low numbers. Overall, the long‐repeat analysis revealed a largely conserved pattern of repeat type and size distribution across the six *Dioscorea* chloroplast genomes, with only minor interspecific differences in repeat abundance.

**FIGURE 5:**
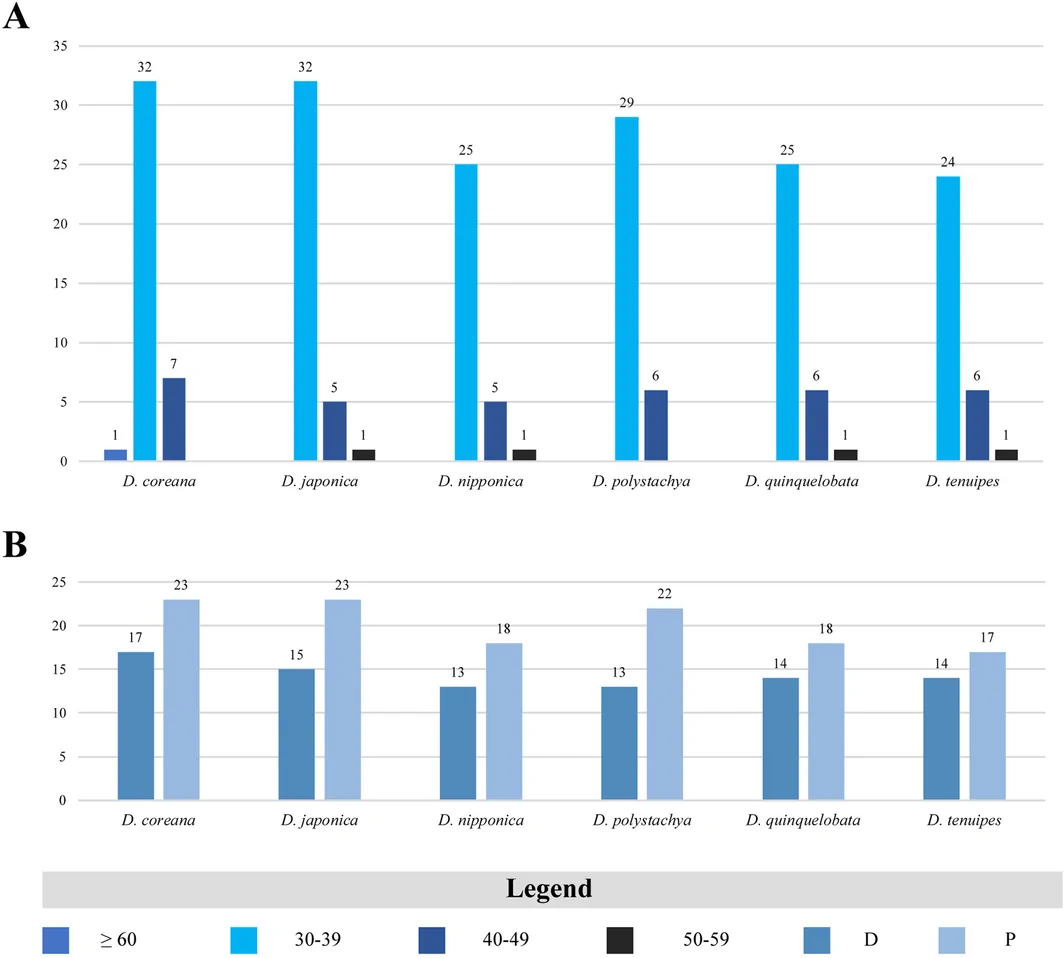
Distribution of long‐repeat sequences in the chloroplast genomes of six *Dioscorea* species. (A) Number of repeat sequences categorized by length interval (30–39, 40–49, 50–59, and ≥ 60 bp). (B) Total counts of repeat types: dispersed (D) and palindromic (P) repeats. The *y*‐axis represents the number of repeat sequences identified in each category.

### 3.4 Localized Sequence Divergence in Dioscorea Chloroplast Genomes

To identify regions of chloroplast genome conservation and divergence among the six *Dioscorea* species, IR boundaries, nucleotide diversity, and sequence divergence were comparatively examined. Size variation in IR regions, caused by expansion and contraction, can lead to structural changes in the chloroplast genome, including differences in gene copy number and pseudogene formation at junction boundaries (Figure 6). In angiosperm chloroplast genomes, IR regions are generally well conserved and contribute to structural stability, whereas expansions and contractions at IR junctions are recognized as important drivers of genome size variation (Li and Zheng 2018). Because such variations can provide informative markers for evolutionary studies (Li et al. 2023; Joh et al. 2025), we examined these regions to characterize patterns of chloroplast genome variation among the six Dioscorea species. The IR regions of the analyzed *Dioscorea* species were highly conserved in both length and gene content, with IR sizes ranging from approximately 25.45 kb to 25.56 kb. All species exhibited a typical gene composition within the IR regions, including duplicated rRNA genes (*rrn16, rrn23, rrn4.5*, and *rrn5*) and several protein‐coding genes, such as *rpl2, rpl23, rps7*, and *ndhB*. Despite this overall conservation, slight variations were observed at the junctions between the IR regions and single‐copy regions. At the IRb/SSC boundary (JSB), partial duplication of the ycf1 gene was consistently detected across all species; however, the length of the duplicated fragment differed slightly among species. Similarly, at the LSC/IRb junction (JLB), small shifts in the position of *rps19* were observed. Some similarities in IR boundary structure were observed among phylogenetically related species, although minor interspecific differences in *ycf1* duplication length and junction positions were also present.

**FIGURE 6:**
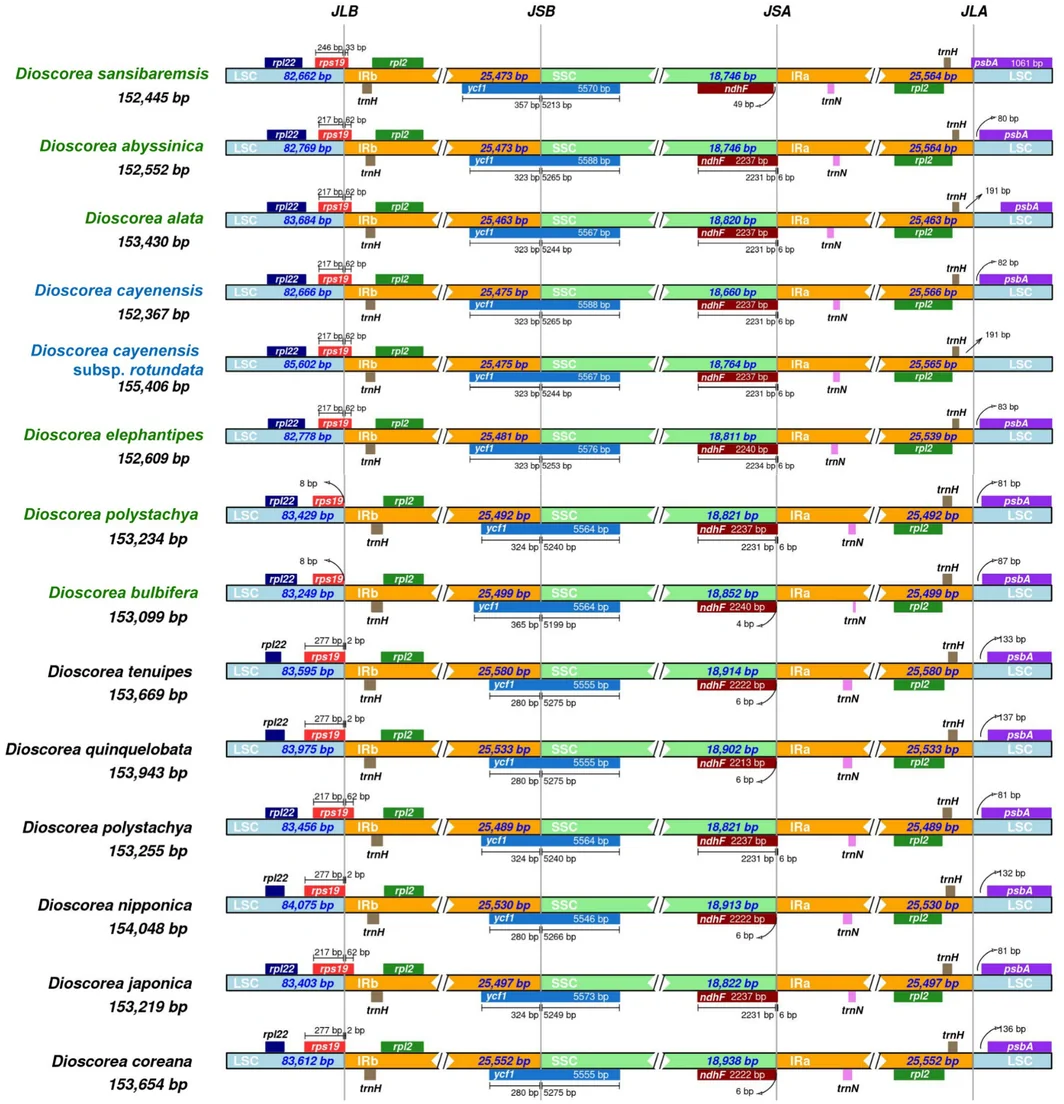
Structural comparison of LSC/IR and SSC/IR junctions across *Dioscorea* chloroplast genomes. Genes flanking each boundary are depicted as boxes, and distances to junction sites are given in base pairs. Terminal labels are color‐coded to represent genetic groups: Black for the six *Dioscorea* species examined in this study, green for landraces, and blue for modern cultivars defined by GRIN–Global.

To identify regions of elevated sequence variation among the six *Dioscorea* chloroplast genomes, π was compared across coding sequences (CDSs) using sliding window analysis (Figure 7). Overall, π values varied across the chloroplast coding regions of the six *Dioscorea* species, although most CDSs remained relatively conserved. Regions exhibiting markedly increased π values (π > 0.04), corresponding to prominent peaks in the distribution, were identified as highly variable loci. Notably, pronounced peaks of variation were observed in specific genes, particularly *ndhF* and *ycf1*, suggesting that these loci represent major hotspots of sequence divergence within the coding regions. In contrast, the majority of CDSs presented low nucleotide diversity, reflecting the generally conserved nature of chloroplast protein‐coding genes. Overall, nucleotide diversity was unevenly distributed across the chloroplast coding regions, with *ndhF* and *ycf1* showing the highest levels of sequence divergence among the examined loci.

**FIGURE 7:**
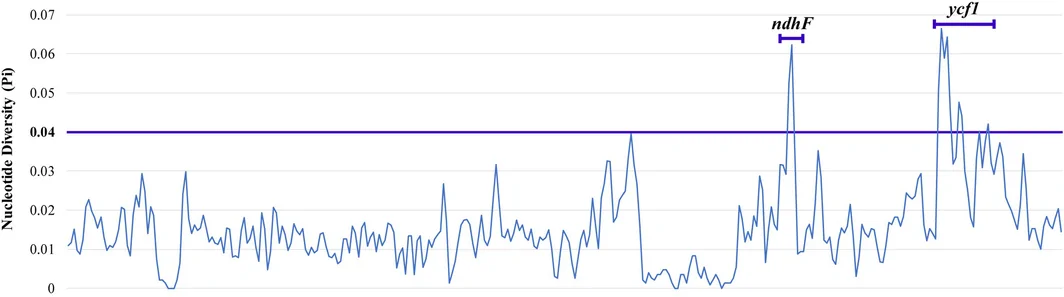
Sliding window analysis of nucleotide diversity (*π*) across concatenated chloroplast protein‐coding sequences of six *Dioscorea* species. The *y*‐axis represents nucleotide diversity values, while the *x*‐axis indicates positions along the concatenated coding‐sequence alignment.

To further characterize patterns of sequence conservation and divergence across the chloroplast genomes, the six *Dioscorea* species were comparatively analyzed using mVISTA, with *D. cayenensis* as the reference (GenBank accession no. MG805606) (Figure 8). The alignment revealed a generally high level of sequence similarity across all the genomes, with conservation patterns corresponding closely to gene organization and orientation indicated in the reference map. Protein‐coding regions were highly conserved among the six species, showing only minor sequence variation. In contrast, conserved noncoding sequences (CNSs), indicated by blue and light blue regions, exhibited relatively high levels of divergence. This pattern was particularly evident in intergenic spacer regions and untranslated regions (UTRs), suggesting that these noncoding regions contribute substantially to genomic variation within the genus. Region‐specific patterns of variation were also observed. The IR regions were strongly conserved with minimal sequence divergence, whereas the LSC region contained the majority of variable sites. The SSC region displayed an intermediate level of variability. Overall, the mVISTA comparison highlights distinct conservation patterns between coding and noncoding regions, with highly conserved gene sequences and more variable intergenic regions.

**FIGURE 8:**
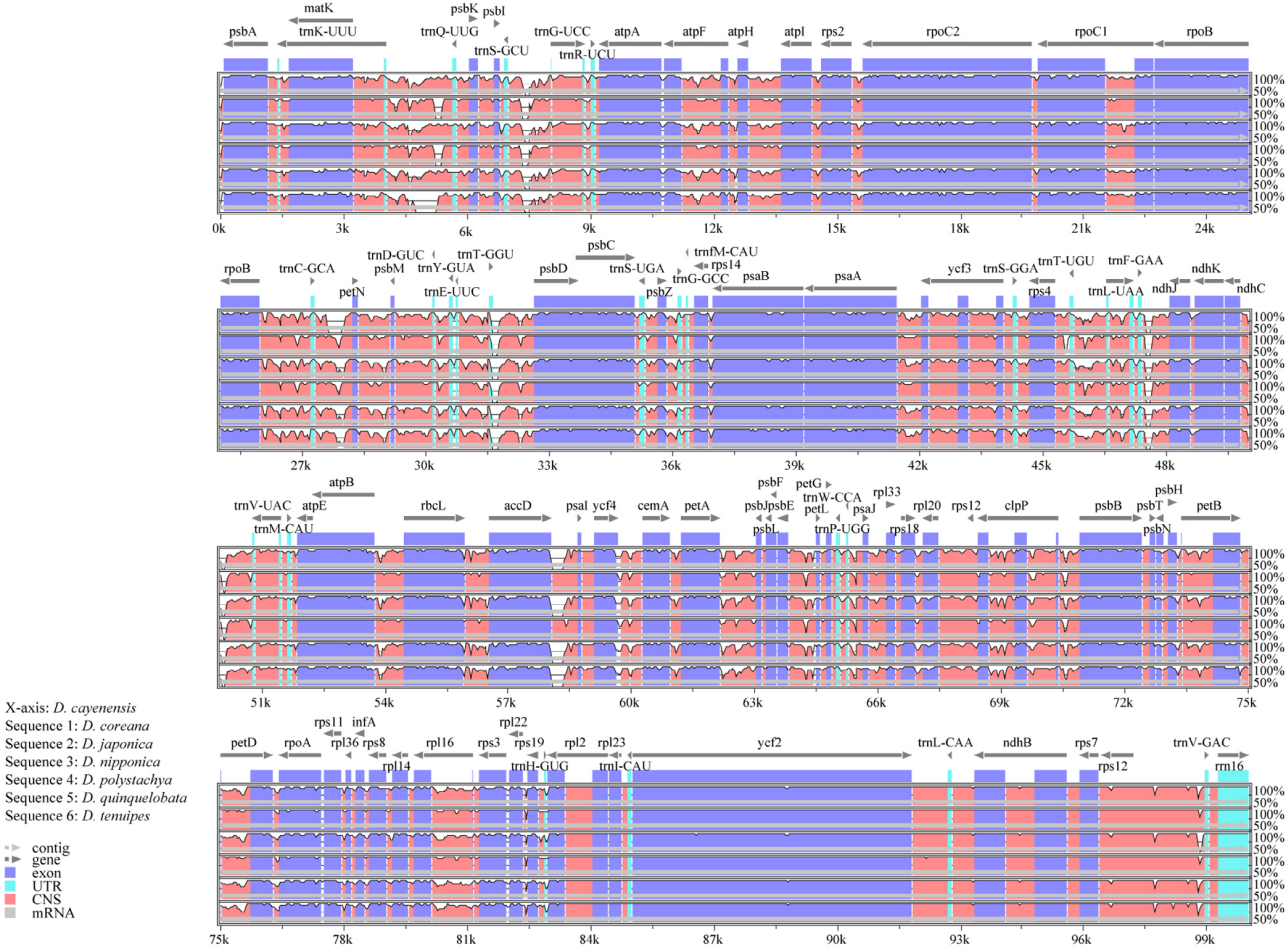
Comparative alignment of six *Dioscorea* chloroplast genomes using mVISTA. *D. cayenensis* (GenBank accession no. MG805606) was used as the reference sequence. Gene orientation and positions are indicated by gray arrows. Protein‐coding regions and conserved CNSs are represented by red, light blue, and blue shading.

### 3.5 Well‐Supported Phylogenetic Relationships Within Dioscorea

To clarify phylogenetic relationships within *Dioscorea* and evaluate the placement of the species analyzed in this study, a ML tree was constructed using concatenated chloroplast protein‐coding gene sequences, with *Tacca* species as outgroups (Figure 9). The phylogenetic tree resolved several well‐supported clades corresponding to recognized taxonomic groupings (Brudno et al. 2003; Hsu et al. 2013; Ngo Ngwe et al. 2015; Couto et al. 2018; Xia et al. 2019). Within the section *Stenophora*, a distinct and well‐supported clade formed, within which the analyzed species (*D. coreana*, *D. quinqueloba*, *D. nipponica* , and *D. tenuipes* ) clustered together with high bootstrap support values. Similarly, species within the section *Enantiophyllum* grouped into a separate clade, indicating clear phylogenetic differentiation among major lineages. In addition, *D. japonica* and *D. polystachya* were positioned within the same clade and showed close phylogenetic relationships, with strong branch support (SH‐aLRT = 99.9%; UFBoot = 100%). Notably, cultivated yam ( *D. polystachya* ) was closely associated with two CWR species, indicating a high degree of genetic proximity. Overall, the tree topology demonstrated strong support at most nodes, highlighting the utility of concatenated chloroplast protein‐coding sequences for resolving interspecific relationships within *Dioscorea*. Taken together, these findings demonstrate overall conservation of chloroplast genome features with localized sequence divergence among the six *Dioscorea* species, providing a genomic framework for interpreting their interspecific relationships.

**FIGURE 9:**
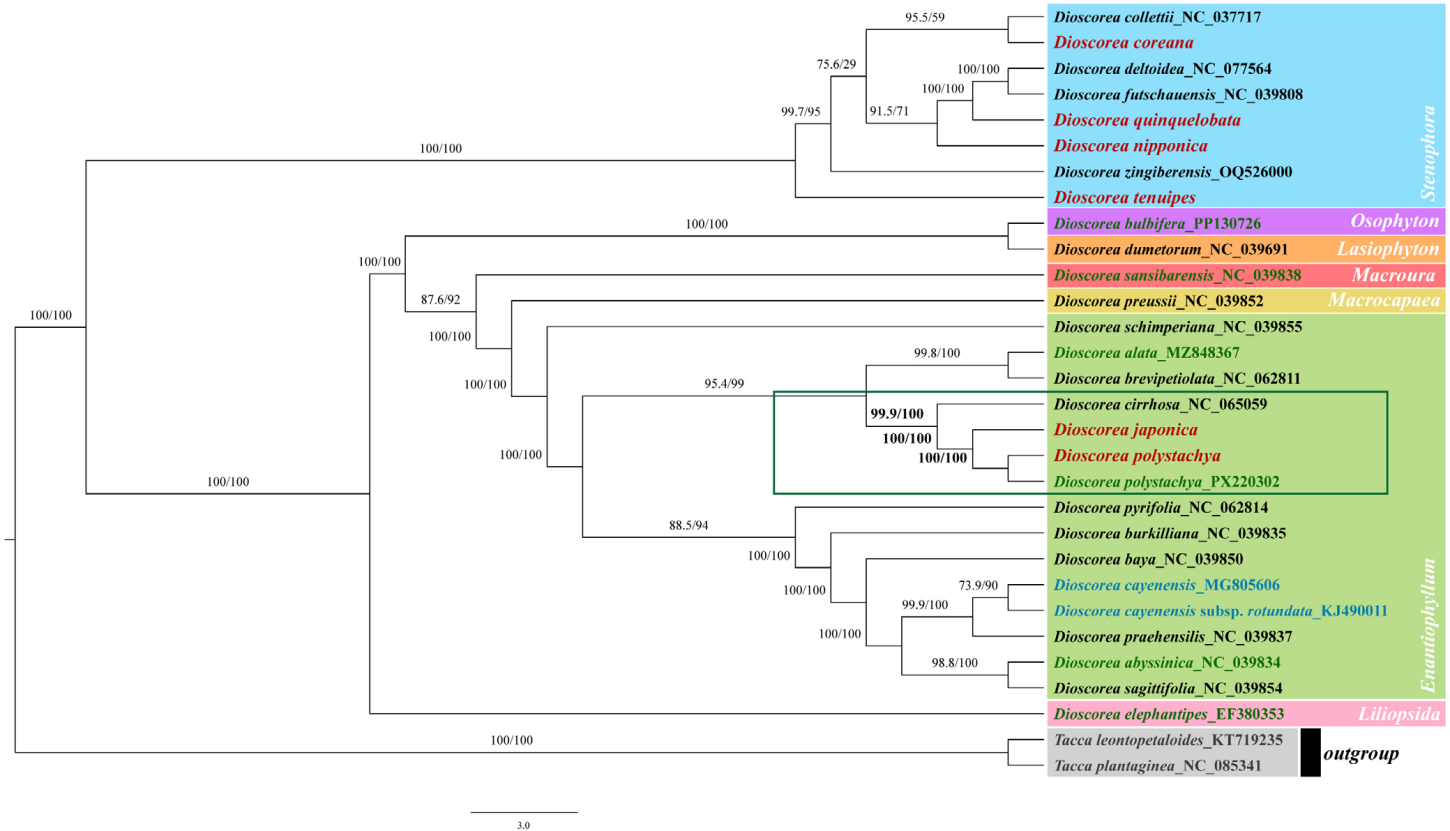
Maximum likelihood tree inferred from concatenated chloroplast protein‐coding gene sequences of *Dioscorea* species. Branch support values are displayed at each node as SH‐aLRT support (%)/ultrafast bootstrap support (%), based on 1000 and 1020 replicates, respectively. Terminal labels are color‐coded to represent genetic groups based on GRIN‐Global distribution information: black for CWRs, green for landraces, red for the six *Dioscorea* species examined in this study, and blue for modern cultivars. The scale bar corresponds to 3.0 substitutions per site.

## 4 Discussion

This study aimed to expand chloroplast genomic information for Korean *Dioscorea* species and to clarify their genomic characteristics and phylogenetic relationships in the context of cultivated yams and their CWRs. Comparative analyses of six complete chloroplast genomes revealed highly conserved genome organization and composition, together with localized sequence divergence in specific coding and noncoding regions. Phylogenetic analysis further resolved well‐supported relationships among major *Dioscorea* lineages and clarified the placement of the Korean taxa examined in this study. Together, these findings expand the genomic resources available for Korean *Dioscorea* and provide a framework for understanding their evolutionary relationships.

At the structural level, the chloroplast genomes of the six Korean *Dioscorea* species showed a high degree of conservation consistent with previously reported genomes within the genus (Cao et al. 2018, 2020; Dai et al. 2025). All six genomes exhibited the canonical quadripartite structure of angiosperm chloroplasts, comprising an LSC region, an SSC region, and a pair of IR regions. The overall GC content (37.01%–37.20%) and the GC distribution pattern, with a higher GC content in the IR regions than in the single‐copy regions, were consistent with the general features of angiosperm chloroplast genomes (Li et al. 2013; Tyagi et al. 2020; Huang et al. 2022). The total number of annotated genes (130) includes duplicated copies located in the IR regions, whereas the number of unique genes is 112. This distinction explains why the total gene count exceeds the approximately 100–120 unique genes typically reported for chloroplast genomes. Notably, in this study, the complete chloroplast genome of *D. coreana* was reported for the first time, offering an important genomic resource for subsequent studies within the genus. Thus, our results extend the chloroplast resources available for Korean *Dioscorea*, particularly for *D. coreana*, while demonstrating the overall conservation of chloroplast genome architecture among the examined taxa.

In addition to the conserved genome structure, codon usage patterns were also highly similar among the six Korean *Dioscorea* species, with a general preference for A/U‐ending synonymous codons (Clegg et al. 1994; Daniell et al. 2016). Similar codon usage trends have been observed across diverse plant lineages, where synonymous codons ending in A or U are generally overrepresented, whereas those ending in G or C are underrepresented (He et al. 2016; Li et al. 2025). This bias is typically attributed to the overall AT‐rich nucleotide composition of chloroplast genomes, particularly at the third codon position, which strongly influences codon selection (Morton 1998). In addition to compositional constraints, both mutational pressure and natural selection have been suggested as major forces that shape codon usage patterns in chloroplast genes (Bulmer 1991; Jenkins and Holmes 2003; Chen et al. 2014; He et al. 2016). Together with previous reports from other angiosperms, the consistent A/U‐ending codon preference observed supports codon usage bias as a conserved feature of chloroplast genomes shaped by nucleotide composition and evolutionary constraints (Daniell et al. 2016).

Consistent with the conserved genome structure and codon usage patterns, repetitive sequences also showed broadly similar characteristics among the six *Dioscorea* chloroplast genomes. The SSR distribution patterns observed in this study are consistent with those reported in angiosperm chloroplast genomes, with a strong predominance of A/T‐rich mononucleotide repeats reflecting the overall AT‐biased composition of the chloroplast genome (Daniell et al. 2016; Song et al. 2022). SSRs were concentrated mainly in the LSC region, whereas the IR regions contained very few repeats, likely because of their conserved nature and copy‐correction mechanisms (Raman et al. 2020). Compared with the LSC and SSC regions, the IR regions generally have a higher GC content, which may limit the formation of A/T‐rich SSR motifs (Moghaddam et al. 2022). In contrast, the LSC region, characterized by relatively lower GC content, provides a more favorable environment for the accumulation of SSRs. Similarly, long‐repeat sequences showed a broadly conserved distribution pattern across the examined species, with palindromic repeats more abundant than dispersed repeats and most repeats falling within the 30–39 bp size range. Although minor differences in repeat abundance were observed among species, the overall similarity further supports the structural conservation of their chloroplast genomes. These findings characterize the conserved repeat landscape of Korean *Dioscorea* chloroplast genomes while identifying interspecific differences that warrant further evaluation for their utility as molecular markers (Pandey and Bhargava 2024; Khade et al. 2025).

Despite this overall conservation, localized sequence variation was evident at IR boundaries and in specific coding and noncoding regions. The slight variations observed at IR junctions, including the partial duplication of *ycf1* and positional shifts of *rps19*, indicate that limited expansion and contraction of IR regions have influenced gene positioning at boundary regions (Zhu et al. 2016; Wonok et al. 2023). Although such dynamics are commonly reported in chloroplast genomes, the changes observed in *Dioscorea* were minor and did not result in substantial structural rearrangements, which is consistent with the conserved IR length and gene content across species (Wonok et al. 2023). This finding suggests that IR regions in *Dioscorea* remain highly stable and may be subject to relatively constrained evolutionary processes. Consistent with this structural conservation, nucleotide diversity across chloroplast coding regions was generally low, reflecting the conserved nature of plastid genes. However, a small number of loci, particularly *ndhF* and *ycf1*, exhibited increased variability, indicating that sequence divergence is concentrated in specific regions (Li et al. 2021; Wonok et al. 2023). Comparative analysis using mVISTA further supported these findings, revealing high conservation in protein‐coding regions and relatively high divergence in noncoding sequences, especially within the LSC and SSC regions, while the IR regions remained highly conserved. Together, these results show that sequence divergence in otherwise conserved *Dioscorea* chloroplast genomes is concentrated in a limited number of coding and noncoding regions. The elevated variation in *ndhF, ycf1*, and several noncoding regions identifies candidate loci for species discrimination and phylogenetic analyses, although their effectiveness as molecular markers requires further validation.

At the phylogenetic level, concatenated chloroplast protein‐coding gene sequences provided strong resolution of relationships among the examined *Dioscorea* taxa. The phylogenetic relationships inferred from concatenated chloroplast protein‐coding gene sequences were largely consistent with previously established infrageneric classifications of *Dioscorea*, supporting the utility of chloroplast coding sequences for phylogenetic reconstruction. In particular, the clear separation of major sections, including *Stenophora* and *Enantiophyllum*, agrees with the results of earlier molecular studies based on chloroplast and nuclear markers, which demonstrated congruent phylogenetic signals across different genomic datasets (Ngo Ngwe et al. 2015; Noda et al. 2022). The formation of a well‐supported clade comprising *D. coreana*, *D. quinqueloba*, *D. nipponica* , and *D. tenuipes* further supports the taxonomic coherence of the *Stenophora* section, which has been recognized as an early‐diverging lineage within the genus. Similarly, the close relationship between *D. japonica* and *D. polystachya* is consistent with the findings of previous phylogenetic studies that resolved morphologically similar taxa as distinct but closely related species (Noda et al. 2022). Furthermore, the phylogenetic relationships resolved in this study revealed that cultivated yam ( *D. polystachya* ) is closely associated with *D. japonica* and *D. polystachya* (in this study), forming a well‐supported clade. These results reinforce the major infrageneric relationships previously proposed for *Dioscorea* while providing additional chloroplast genomic evidence for the phylogenetic placement of the Korean taxa examined here.

Beyond clarifying taxonomic relationships, the phylogenetic framework established here also provides a basis for evaluating Korean wild *Dioscorea* species in the context of CWR conservation and utilization. Wild relatives distributed across diverse ecological environments may harbor adaptive traits associated with abiotic stress tolerance, disease resistance, and environmental resilience, which are becoming increasingly important under changing climatic conditions. Although adaptive and agronomic traits were not directly evaluated in this study, the phylogenetic framework provides an initial basis for prioritizing Korean *Dioscorea* taxa for subsequent functional evaluation. However, phylogenetic proximity alone should not be interpreted as evidence of breeding value, which requires direct assessment of phenotypic traits, genetic diversity, and cross‐compatibility. This information may facilitate the selection of relevant wild relatives for future studies on adaptive traits, including those associated with environmental stress tolerance, and support subsequent efforts in molecular marker development and crop improvement under changing environmental conditions (Mukherjee and Bhat 2013; Magwé‐Tindo et al. 2018). Accordingly, the relationships resolved here provide a genomic basis for prioritizing candidate Korean *Dioscorea* CWRs for conservation and further assessment, while direct evaluation of adaptive and agronomic traits will be required to establish their value for crop improvement.

## 5 Conclusion

This study expands the chloroplast genomic resources available for Korean Dioscorea and provides a comparative framework for placing these taxa within the broader evolutionary context of cultivated yams and their wild relatives. Beyond improving genomic representation of Korean taxa, these resources can support the prioritization of potential CWRs for conservation and subsequent evaluation. However, phylogenetic proximity alone does not establish the agronomic or breeding value of wild relatives. Integrating chloroplast and nuclear genomic data with population‐level variation, phenotypic traits, and breeding compatibility will be important for translating the genomic relationships identified here into practical strategies for germplasm conservation and yam improvement.

## Author Contributions

**Ji Eun Kim:** conceptualization (equal), data curation (lead), formal analysis (equal), investigation (lead), methodology (lead), visualization (lead), writing – original draft (lead). **Min Su Jo:** conceptualization (equal). **Kyung‐Bae Nam:** conceptualization (lead). **Jun Hyeok Kim:** conceptualization (supporting), project administration (equal). **Jun‐Ho Song:** resources (supporting). **Hyeok‐Jae Choi:** resources (supporting). **Gyu Young Chung:** resources (lead). **Chae Sun Na:** project administration (lead), writing – original draft (supporting), writing – review and editing (lead).

## Funding

This research was funded by the “R&D Program for Forest Science Technology (project no. RS‐2021‐KF001796)” supported by the Korea Forest Service (Korea Forestry Promotion Institute).

## Ethics Statement

The authors have nothing to report.

## Conflicts of Interest

The authors declare no conflicts of interest.

## Data Availability

The six complete chloroplast genome sequences generated in this study have been deposited in the NCBI GenBank database under accession numbers PZ211770–PZ211775. The corresponding records are also available through the Korea Biodiversity Data System (K‐BDS), under accession numbers SN01002270–SN01002275.
